# Star Image Registration Modeling and Parameter Calibration for a 3CMOS Star Sensor

**DOI:** 10.3390/s24010259

**Published:** 2024-01-02

**Authors:** Yanzhao Niu, Xinguo Wei, Jian Li

**Affiliations:** Key Laboratory of Precision Opto-Mechatronics Technology, Ministry of Education, School of Instrumentation Science and Opto-Electronics Engineering, Beihang University, 37 Xueyuan Rd., Haidian District, Beijing 100191, China; nyz2011@126.com (Y.N.); lijian_0355@163.com (J.L.)

**Keywords:** star sensor, imaging modeling, star image registration, parameter calibration

## Abstract

This paper presents an image registration method specifically designed for a star sensor equipped with three complementary metal oxide semiconductor (CMOS) detectors. Its purpose is to register the red-, green-, and blue-channel star images acquired from three CMOS detectors, assuring the precision of star image fusion and centroid extraction in subsequent stages. This study starts with a theoretical analysis aimed at investigating the effect of inconsistent three-channel imaging parameters on the position of feature points. Based on this analysis, this paper establishes a registration model for transforming the red- and blue-channel star images into the green channel’s coordinate system. Subsequently, the method estimates model parameters by finding a nonlinear least-squares solution. The experimental results demonstrate the correctness of the theoretical analysis and the proposed registration method. This method can achieve subpixel alignment accuracy in both the x and y directions, thus effectively ensuring the performance of subsequent operation steps in the 3CMOS star sensor.

## 1. Introduction

As a crucial attitude measurement device, a star sensor [1,2] uses stars as references to determine the three-axis attitude without a priori information. With the characteristics of high precision, autonomous sensing, and no time drift, star sensors have been widely utilized in various spacecraft attitude control systems. The performance of a star sensor is mainly determined by fast and robust star identification and highly accurate attitude estimates.

Current strategies for fast and robust star identification depend on detecting more stars to construct a unique feature pattern [3]. Traditionally, this is achieved by expanding the field of view (FOV) or observing dimmer stars, but these approaches involve many interesting trade-offs in the optical design of a star sensor [4,5]. Alternatively, researchers have explored methods to enhance star identification performance by extracting new information from individual stars. Quine [6] introduced stellar magnitude information, but accurately obtaining it using existing star sensor optical systems is a challenge [7]. Enright and Mcvittie [8] proposed combining the extracted color information and angular distance of two stars into a 1D Mahalanobis distance as the matching element for star identification; this research proves that color information can effectively reduce the number of stars required for star identification. Furthermore, Niu et al. [9] employed star color information as an independent matching feature to further enhance star identification robustness to position noise. Their studies demonstrate that star color information can be used for fast and robust star identification, even in challenging conditions, such as in sky regions with fewer detectable stars or higher-level position noise in highly dynamic scenarios [10]. The application of star color information provides a novel solution to enhance the performance of star sensors. More importantly, these studies also suggest that the more accurate the color information obtained, the greater the improvement in star identification performance.

Studies on obtaining color information from a star can be divided into two main categories: color filter array (CFA) detectors and 3CMOS detectors. CFAs employ different filters on each pixel to accept specific bandpass signals. However, this method comes with the drawback of intensity loss [11]. Additionally, due to the sub-sampling of a CFA, this technology causes high noise when measuring star color information [12]. In contrast, a 3CMOS detector system overcomes these limitations by using a prism beam splitting system that separates the incident starlight between particular detectors depending on the wavelength. Each detector observes a specific color of the image, generating full-frame images with a resolution equivalent to that of monochromatic star sensors while effectively avoiding intensity loss. As stated in study [12], 3CMOS imaging outperforms CFA imaging in providing accurate star color information, making it a superior method for capturing star color information. When this imaging technology is applied to a star sensor, it is referred to as a 3CMOS star sensor.

During the operation of a 3CMOS star sensor, obtaining a fusion complementary star image is crucial for accurately extracting star centroids, ensuring the accuracy of final attitude measurements. Image fusion in the multi-sensor workflow typically involves preprocessing, feature extraction, registration, and decision fusion. While the preprocessing and feature extraction may vary depending on the specific application, image registration [13] plays a critical step in the fusion process. It involves aligning images of the same target captured in different spatial coordinates to a reference system, significantly impacting the performance of subsequent steps. Due to factors such as chromatic aberrations and inherent detector alignment errors, misalignment between the three star images captured from three detectors is inevitable in the 3CMOS star sensor. A previous work [12] simply fused three star images without considering the crucial step of image registration. As a high-precision attitude measurement component, the three star images must be registered before fusing them. In response to this, this paper proposes a star image registration method based on the geometric imaging properties of 3CMOS star sensors. The study mainly includes constructing registration models and conducting high-precision parameter calibration. The remainder of this paper is structured as follows. Section 2 details the imaging and transformation modeling process. Section 3 explains the model parameter-solving process, and Section 4 presents the results of experiments conducted to evaluate the proposed method. Finally, the paper concludes with a summary of the main findings and directions for future research.

## 2. Model Description

**Figure 1:**
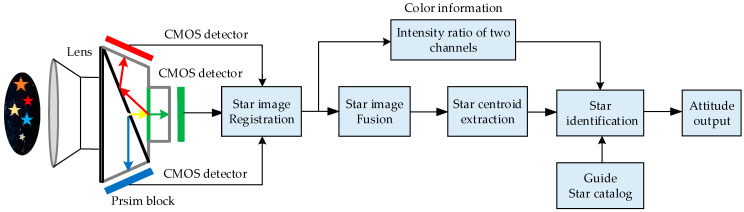
Working flowchart of 3CMOS star sensor.

Stars are divided into different spectral types [14], each of which is associated with unique radiation properties and a specific color representing its peak radiation [15]. Traditional star sensors rely solely on star position information extracted from a single monochromatic detector [16] to provide attitude solutions. In contrast, a 3CMOS star sensor uses a prism imaging system to split the incident light of stars into red, green, and blue color channels based on the wavelength, as shown in Figure 1. The separated starlight is then directed to its respective detector, forming a star spot.

For a given observed star, the intensity of the star spots imaged by three detectors is different due to the star’s spectral emissivity changing with wavelength. Additionally, the intensity ratio of stars also changes with the randomly distributed spectral types within the FOV. Therefore, a 3CMOS star sensor defines the intensity ratio [8,9] of its two channels as the color information for an observed star and uses it to enhance the star identification performance. Following the workflow illustrated in Figure 1, this study primarily focuses on the accurate registration of star images obtained by three detectors to lay a foundation for subsequent accurate star image fusion and star centroid extraction, thereby ensuring the final attitude measurement’s accuracy.

To achieve this goal, we take the position of imaging star spots as feature points. Based on the established single-channel imaging models, we conduct a comprehensive analysis of the impact of inconsistent three-channel imaging parameters on the position of feature points and then derive the star image registration model.

### 2.1. Single-Channel Imaging Modeling

During the imaging process of a 3CMOS star sensor, the incident starlight is separated and directed to the respective channel, and a pinhole model [1,16,17] can be used to approximate single-channel imaging. Taking the green channel as an example, its frame can be denoted as $O_{g}-X_{g}Y_{g}Z_{g}$, and the *x*-axis ($X_{g}$) and *y*-axis ($Y_{g}$) are the row and column of the image sensor plane, respectively. The *z*-axis ($Z_{g}$) is normal to the image sensor plane.

For a given observed star, the ideal position of the green-channel imaging star spot, denoted as $\left(\overset{\prime }{x_{g}},\overset{\prime }{y_{g}}\right)$, can be determined using the following expression based on the principle of perspective projection

(1) $$\left\{\begin{array}{c} \overset{\prime }{x_{g}}=F_{g}\cdot \frac{v\left(1\right)}{v\left(3\right)}\\ \overset{\prime }{y_{g}}=F_{g}\cdot \frac{v\left(2\right)}{v\left(3\right)} \end{array}\right.,$$

where **v** represents the incident starlight vector of the observed star, and $F_{g}$ is the imaging focal length of the green channel. However, optical lens distortion results in an offset between the real spot position $\left(x_{g},y_{g}\right)$ and the ideal spot position $\left(\overset{\prime }{x_{g}},\overset{\prime }{y_{g}}\right)$. The deviations in the X and Y directions are denoted as $\left(\delta x_{g},\delta y_{g}\right)$. Under these conditions, the real position of the green-channel imaging spot $\left(x_{g},y_{g}\right)$ can be expressed as

(2) $$\left\{\begin{array}{c} x_{g}=\overset{\prime }{x_{g}}+x_{0}^{g}+\delta x_{g}\\ y_{g}=\overset{\prime }{y_{g}}+y_{0}^{g}+\delta y_{g} \end{array}\right.,$$

where ${(x}_{0}^{g},y_{0}^{g})$ is the coordinate of the principal point, which is the intersection of the boresight and the detector image and can be denoted as origin $O_{g}$. Lens distortion can be categorized into radial distortion and tangential distortion [18], each of which requires an infinite series. Tsai [17] noted that only radial distortion needs to be considered, and only the first term is needed; more elaborate modeling not only would not help but would also cause numerical instability. Meanwhile, Luhmann [19] revealed that approximately 90% of the distortion effect can be typically modeled by the first-order radial distortion coefficient. Research [20,21,22,23] further confirmed that the distortion function is mainly dominated by the radial component and especially dominated by the first term. Therefore, to reduce the computational complexity, only the first-term distortion is employed to describe the distortion in this study; the deviations $\left(\delta x_{g},\delta y_{g}\right)$ are expressed as

(3) $$\left\{\begin{array}{c} \delta x_{g}=\overset{\prime }{x_{g}}m_{g}({\overset{\prime }{x_{g}}}^{2}+{\overset{\prime }{y_{g}}}^{2})\\ \delta y_{g}=\overset{\prime }{y_{g}}m_{g}({\overset{\prime }{x_{g}}}^{2}+{\overset{\prime }{y_{g}}}^{2}) \end{array}\right.,$$

where $m_{g}$ represents the first-order radial distortion coefficient. In this established single-channel imaging model as described in Equation (2), the focal length $F_{g}$, principal point ${(x}_{0}^{g},y_{0}^{g})$ and distortion coefficient $m_{g}$ are defined as imaging parameters. Following this definition, the positions of star spots in the red and blue imaging channels for the same observed star can be determined and expressed as $\left(x_{r},y_{r}\right)$ and $\left(x_{b},y_{b}\right)$, respectively.

### 2.2. Analysis of Differences in Three-Channel Imaging

In the case of a 3CMOS star sensor, the incoming light from one observed star is separated into red, green, and blue channels based on wavelength. Simultaneously, the star spot positions of the three channels are extracted from three separate detectors. Consequently, differences in imaging parameters among the three channels are inevitable due to two primary factors: (1) differences in the imaging wavelengths of the three channels; (2) inherent alignment errors in the positions of the three detectors.

Firstly, due to the sensitivity of the refractive index of transparent materials (such as the glass in a lens) to the wavelength of light, chromatic aberrations can occur in the imaging process of 3CMOS star sensors. These chromatic aberrations manifest as longitudinal chromatic aberrations and transversal chromatic aberrations. Longitudinal chromatic aberrations describe changes in the imaging focal length with wavelength. Furthermore, different imaging wavelengths can also lead to changes in the distortion coefficient, as discussed in [24], which can be considered as a type of transversal chromatic aberrations. Consequently, chromatic aberrations introduce inconsistency in both the imaging focal length and radial distortion coefficient between the three channels. According to Equations (1) and (3), this difference in the imaging parameter can introduce a scaling relationship between the positions of the imaging star spot between three channels when observing the same star.

Secondly, the inherent alignment errors among the three detectors lead to translations and rotations between the image plane, the translations will result in inconsistency in the principal points across the three channels, while the rotations cause a positional shift among the imaging star spots across the three channels when observing the same star.

Based on the above analysis, an affine model was employed to describe the relationship between the positions of three-channel imaging star spots for a given observed star. For instance, the relationship between the green channel’s imaging star spot position $\left(x_{g},y_{g}\right)$ and the blue channel’s imaging star spot position $\left(x_{b},y_{b}\right)$ can be described as

(4) $$\left(\begin{array}{c} x_{g}\\ y_{g} \end{array}\right)=T\cdot \left(\begin{array}{c} x_{b}\\ y_{b} \end{array}\right)+\left(\begin{array}{c} ∆x\\ ∆y \end{array}\right),$$

where **T** is the matrix that describes rotation and scaling, and $\left(∆x,∆y\right)$ describes translation. Based on Equation (4), the detailed transformation model was derived to achieve the registration of star images.

### 2.3. Star Image Coordinate Transformation Relationship Modeling

In this model, the green-channel imaging frame $O_{g}-X_{g}Y_{g}Z_{g}$, situated at the middle wavelength range, was chosen as the reference frame to minimize the effects of distortion. We developed the transformation model by analyzing the influence of focal length, distortion, rotation, and translation on the position of stars imaged in three channels. The transformation of star spot position from the blue-channel coordinate system to the green channel’s imaging coordinate system was taken as an example to show the detailed modeling process.

#### 2.3.1. Case of Focal Length and Distortion

This analysis begins by assuming that there are no rotation and translation phenomena between the blue-channel image plane and the green-channel image. At this point, we assume that the focal length in the blue-channel imaging coordinate $O_{b}-X_{b}Y_{b}Z_{b}$ is expressed as $F_{b}$. Given the same observed star, the ideal position of the blue-channel imaging star spot $\left(\overset{\prime }{x_{b}},\overset{\prime }{y_{b}}\right)$ can be expressed as

(5) $$\left\{\begin{array}{c} \overset{\prime }{x_{b}}=F_{b}\cdot \frac{v\left(1\right)}{v\left(3\right)}\\ \overset{\prime }{y_{b}}=F_{b}\cdot \frac{v\left(2\right)}{v\left(3\right)} \end{array}\right.,$$

Noting $k_{bg}=F_{g}/F_{b}$, we can obtain the ratio relationship between the ideal position of the green-channel imaging star spot $\left(\overset{\prime }{x_{g}},\overset{\prime }{y_{g}}\right)$ and the ideal position of the blue-channel imaging star spot $\left(\overset{\prime }{x_{b}},\overset{\prime }{y_{b}}\right)$, which is expressed as

(6) $$\left\{\begin{array}{c} \overset{\prime }{x_{g}}=k_{bg}\cdot \overset{\prime }{x_{b}}\\ \overset{\prime }{y_{g}}=k_{bg}\cdot \overset{\prime }{y_{b}} \end{array}\right.,$$

Meanwhile, substituting Equation (6) into the expression for green-channel distortion, as shown in Equation (3), allows us to derive the ratio relationship between distortion in the green channel $\left(\delta x_{g},\delta y_{g}\right)$ and in the blue channel $\left(\delta x_{b},\delta y_{b}\right)$, shown as

(7) $$\left\{\begin{array}{c} k_{\delta x,bg}=\frac{\delta x_{g}}{\delta x_{b}}=\frac{\overset{\prime }{x_{g}}m_{g}\left({\overset{\prime }{x_{g}}}^{2}+{\overset{\prime }{y_{g}}}^{2}\right)}{\overset{\prime }{x_{b}}m_{b}\left({\overset{\prime }{x_{b}}}^{2}+{\overset{\prime }{y_{b}}}^{2}\right)}=\frac{k_{bg}^{3}\cdot \overset{\prime }{x_{b}}\cdot m_{g}\left({\overset{\prime }{x_{b}}}^{2}+{\overset{\prime }{y_{b}}}^{2}\right)}{\overset{\prime }{x_{b}}\cdot m_{b}\left({\overset{\prime }{x_{b}}}^{2}+{\overset{\prime }{y_{b}}}^{2}\right)}=\frac{k_{bg}^{3}\cdot m_{g}}{m_{b}}\\ k_{\delta y,bg}=\frac{\delta y_{g}}{\delta y_{b}}=\frac{\overset{\prime }{y_{g}}m_{g}\left({\overset{\prime }{x_{g}}}^{2}+{\overset{\prime }{y_{g}}}^{2}\right)}{\overset{\prime }{y_{g}}m_{b}\left({\overset{\prime }{x_{b}}}^{2}+{\overset{\prime }{y_{b}}}^{2}\right)}=\frac{k_{bg}^{3}\cdot \overset{\prime }{y_{b}}\cdot m_{g}\left({\overset{\prime }{x_{b}}}^{2}+{\overset{\prime }{y_{b}}}^{2}\right)}{\overset{\prime }{y_{b}}\cdot m_{b}\left({\overset{\prime }{x_{b}}}^{2}+{\overset{\prime }{y_{b}}}^{2}\right)}=\frac{k_{bg}^{3}\cdot m_{g}}{m_{b}} \end{array}\right.,$$

For a 3CMOS star sensor, variations in the first-order distortion coefficient among the three channels primarily stem from differences in wavelength. Klein et al. [24] revealed that when the imaging wavelength is changed from 400 nm to 650 nm, the change in the first-order radial coefficient is very small, approximately 3.78 × 10^−3^. For the 3CMOS star sensor, the center wavelengths of the three-channel imaging are 450 nm, 550 nm, and 650 nm, respectively. Therefore, to simplify analysis and facilitate star image registration, the distortion coefficients $m_{r}{,\;m}_{g}$, $m_{b}$ were considered to be approximately equal in this model. Under this condition, Equation (7) can be converted into the following expression

(8) $$\left\{\begin{array}{c} k_{\delta x,bg}=\frac{\delta x_{g}}{\delta x_{b}}=k_{bg}^{3}\\ k_{\delta y,bg}=\frac{\delta y_{g}}{\delta y_{b}}=k_{bg}^{3} \end{array}\right.,$$

In practice situations, after the assembly of a 3CMOS star sensor, the focal length changes between the three channels caused by chromatic aberration are relatively small due to the close proximity of the imaging wavelengths. Thus, the value of $k_{bg}$ can be reasonably approximated as 1. Under these conditions, it is justifiable to assume that $k_{bg}^{3}$ and $k_{bg}$ are roughly equivalent. Then, the distortion relationship between the green channel and blue channel can be approximately described as

(9) $$\left\{\begin{array}{c} \delta x_{g}=k_{bg}^{3}\cdot \delta x_{b}\approx k_{bg}\cdot \delta x_{b}\\ \delta y_{g}=k_{bg}^{3}\cdot \delta y_{b}\approx k_{bg}\cdot \delta y_{b} \end{array}\right.,$$

The principal point of the star sensor is defined as the intersection of the boresight and the detection image plane, and changes in focal length do not affect the position of the principal point. Under this condition, by substituting Equations (5) and (9) into the green-channel imaging model described as Equation (2), the relationship between the actual position of the green-channel imaging spot $\left(x_{g},y_{g}\right)$, the ideal position of the blue-channel imaging spot $\left(\overset{\prime }{x_{b}},\overset{\prime }{y_{b}}\right)$, blue-channel distortion $\left(\delta x_{b},\delta y_{b}\right)$, and the blue-channel principal point ${(x}_{0}^{b},y_{0}^{b})$ can be expressed as

(10) $$\left\{\begin{array}{c} x_{g}=k_{bg}\cdot \overset{\prime }{x_{b}}+k_{bg}\cdot \delta x_{b}+x_{0}^{b}\\ y_{g}=k_{bg}\cdot \overset{\prime }{y_{b}}+k_{bg}\cdot \delta y_{b}+y_{0}^{b} \end{array}\right.,$$

According to the single-channel imaging model described in Equation (2), we can obtain

(11) $$\left\{\begin{array}{c} \overset{\prime }{x_{b}}+\delta x_{b}=x_{b}-x_{0}^{b}\\ \overset{\prime }{y_{b}}+\delta y_{b}=y_{b}-y_{0}^{b} \end{array}\right.,$$

By substituting Equation (11) into Equation (10), the functional relationship between the position of the green-channel imaging spot $\left(x_{g},y_{g}\right)$ and the position of the blue-channel imaging star spot $\left(x_{b},y_{b}\right)$, considering only focal length and distortion, can be expressed as

(12) $$\left\{\begin{array}{c} x_{g}=k_{bg}\cdot x_{b}-k_{bg}\cdot x_{0}^{b}+x_{0}^{b}\\ y_{g}=k_{bg}\cdot y_{b}-k_{bg}\cdot y_{0}^{b}+y_{0}^{b} \end{array}\right.,$$

#### 2.3.2. The Case of Image Plane Rotation and Translation

**Figure 2:**
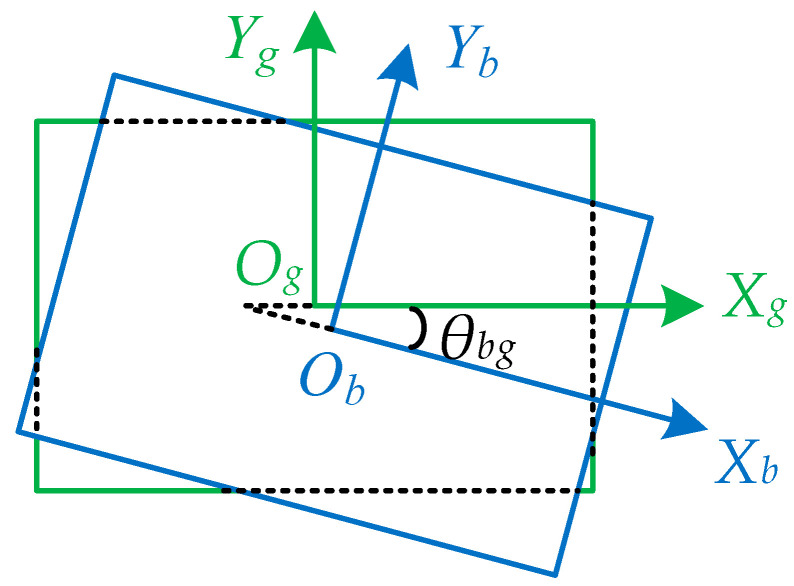
Rotation and translation of the blue-channel image plane relative to the green-channel image plane.

For 3CMOS star sensors, the tilt of the detector’s image plane can be analyzed and corrected in a manner similar to traditional star sensors [16,25]. This model neglects this factor for simplification. Therefore, image plane rotation can be considered to occur around its own optical axis. Taking the green-channel image plane as a reference, the relative position relationship between the blue-channel image planes and green-channel image planes is shown in Figure 2.

Assuming that the rotation angle of the blue-channel image plane relative to the green-channel image plane coordinate system is $\theta_{bg}$, based on Equation (12), the rotation relationship between the position of the green-channel imaging star spot $\left(x_{g},y_{g}\right)$ and the position of the blue-channel imaging star spot $\left(x_{b},y_{b}\right)$ can be expressed as

(13) $$\left[\begin{array}{c} x_{g}\\ y_{g} \end{array}\right]=k_{bg}\left[\begin{array}{cc} \cos\theta_{bg} & -\sin\theta_{bg}\\ \sin\theta_{bg} & \cos\theta_{bg} \end{array}\right]\left[\begin{array}{c} x_{b}-x_{0}^{b}\\ y_{b}-y_{0}^{b} \end{array}\right]+\left[\begin{array}{c} x_{0}^{b}\\ y_{0}^{b} \end{array}\right],$$

Furthermore, assuming that a relative translation between the blue-channel image plane and the green-channel image plane is denoted as ($∆x_{bg},∆y_{bg}$), this translation of the image plane will change the principal point where the optical axis intersects the image plane, leading to an overall shift of the imaging star spot position coordinates. Thus, we can take this translation as an offset of the principal point and express it as

(14) $$\left\{\begin{array}{c} ∆x_{bg}=x_{0}^{b}-x_{0}^{g}\\ ∆y_{bg}=y_{0}^{b}-y_{0}^{g} \end{array}\right.,$$

According to the channel imaging model described in Equation (2), incorporating Equation (14) into Equation (13) yields the final transformation relationship between the position of the green-channel imaging star spot $\left(x_{g},y_{g}\right)$ and the position of the blue-channel imaging star spot $\left(x_{b},y_{b}\right)$, expressed as

(15) $$\left[\begin{array}{c} x_{g}\\ y_{g} \end{array}\right]=k_{bg}\left[\begin{array}{cc} \cos\theta_{bg} & -\sin\theta_{bg}\\ \sin\theta_{bg} & \cos\theta_{bg} \end{array}\right]\left[\begin{array}{c} x_{b}-x_{0}^{b}\\ y_{b}-y_{0}^{b} \end{array}\right]+\left[\begin{array}{c} x_{0}^{b}\\ y_{0}^{b} \end{array}\right]-\left[\begin{array}{c} ∆x_{bg}\\ ∆y_{bg} \end{array}\right],$$

After sorting, Equation (15) can be converted into

(16) $$\left[\begin{array}{c} x_{g}\\ y_{g} \end{array}\right]=k_{bg}\left[\begin{array}{cc} \cos\theta_{bg} & -\sin\theta_{bg}\\ \sin\theta_{bg} & \cos\theta_{bg} \end{array}\right]\left[\begin{array}{c} x_{b}\\ y_{b} \end{array}\right]-k_{bg}\left[\begin{array}{cc} \cos\theta_{bg} & -\sin\theta_{bg}\\ \sin\theta_{bg} & \cos\theta_{bg} \end{array}\right]\left[\begin{array}{c} x_{0}^{b}\\ y_{0}^{b} \end{array}\right]+\left[\begin{array}{c} x_{0}^{b}\\ y_{0}^{b} \end{array}\right]-\left[\begin{array}{c} ∆x_{bg}\\ ∆y_{bg} \end{array}\right],$$

As can be seen from Equation (16), the position of the star spot in the blue-channel coordinate system can be transformed into the green-channel coordinate system through scaling, rotating, and translating operations, thus enabling the registration of the star images for the 3CMOS star sensor. To facilitate analysis, Equation (16) is converted into the following expressions

(17) $$\;\;\left\{\begin{array}{c} x_{g}=k_{bg}\cdot \cos\theta_{bg}\cdot x_{b}-k_{bg}\cdot \sin\theta_{bg}\cdot y_{b}-k_{bg}\cdot \cos\theta_{bg}\cdot x_{0}^{b}+k_{bg}\cdot \sin\theta_{bg}\cdot y_{0}^{b}+x_{0}^{b}-∆x_{bg}=f_{x}(u_{bg})\\ y_{g}=k_{bg}\cdot \sin\theta_{bg}\cdot x_{b}+k_{bg}\cdot \cos\theta_{bg}\cdot y_{b}-k_{bg}\cdot \sin\theta_{bg}\cdot x_{0}^{b}-k_{bg}\cdot \cos\theta_{bg}\cdot y_{0}^{b}+y_{0}^{b}-∆y_{bg}=f_{y}(u_{bg}) \end{array}\right.,$$

where $u_{bg\;}$ is denoted as the parameter vector consisting of four parameters ($k_{bg},\theta_{bg},∆x_{bg},\;∆y_{bg}$). Similarly, the transformation relationship between the star spot centroid $\left(x_{r},y_{r}\right)$ in the red-channel coordinate system and the star spot centroid $\left(x_{g},y_{g}\right)$ in the green-channel coordinate system can be expressed as follows

(18) $$\left\{\begin{array}{c} x_{g}=k_{rg}\cdot \cos\theta_{rg}\cdot x_{r}-k_{rg}\cdot \sin\theta_{rg}\cdot y_{r}-k_{rg}\cdot \cos\theta_{rg}\cdot x_{0}^{r}+k_{rg}\cdot \sin\theta_{rg}\cdot y_{0}^{r}+x_{0}^{r}-∆x_{rg}=f_{x}(u_{rg})\\ y_{g}=k_{rg}\cdot \sin\theta_{rg}\cdot x_{r}+k_{rg}\cdot \cos\theta_{rg}\cdot y_{r}-k_{rg}\cdot \sin\theta_{rg}\cdot x_{0}^{r}-k_{rg}\cdot \cos\theta_{rg}\cdot y_{0}^{r}+y_{0}^{r}-∆y_{rg}=f_{y}(u_{rg}) \end{array}\right.,$$

where the parameter vectors $u_{rg}$ also comprises four parameters $\left(k_{rg},\theta_{rg},∆x_{rg},∆y_{rg}\right)$. Hence, there are eight model parameters are extracted in this transformation model to register the three star images for the 3CMOS star sensor.

## 3. Parameter Calibration

Once the 3CMOS star sensor assembly was complete, the corresponding focal lengths, rotation, and translation relationships between the three detectors were determined. To achieve precise star image registration, accurate calibration of the model parameters was required. To this end, the calibration process for the parameter vectors of $u_{bg\;}$ and $u_{rg}\;$ was carried out in the following steps.

### 3.1. Calibration Data Acquisition

**Figure 3:**
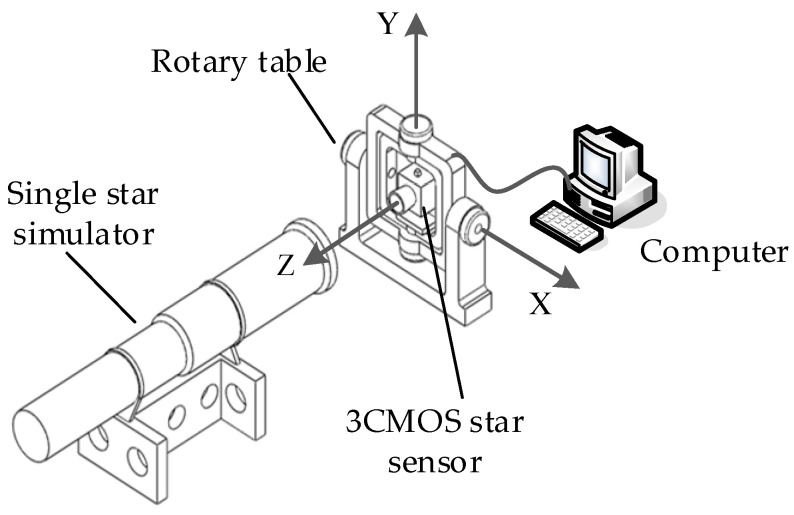
Calibration system configuration for 3CMOS star sensor.

An number of calibration data were collected using the setups depicted shown in Figure 3. A single star simulator with an ultralong focus emitted parallel light, similar to real starlight, while a high-precision rotary table was used to generate different angles. The 3CMOS star sensor was mounted on the middle frame, with its boresight pointing towards the star simulator. During the calibration process, the computer recorded both the rotation angle of the external and middle frames of the rotary table, as well as the centroid coordinates of the star spot on three detectors. The subpixel subdivision positioning algorithm [26] was used to calculate the centroid coordinate of each star spot.

The value of the model parameters was solely determined by the relative installation position of the three detectors. For convenience in calculation, one suitable initial position is to align the simulated starlight vector with the *z*-axis of the 3CMOS star sensor, with minimum error. In this case, the direction of the simulated starlight vector in the coordinate system of the 3CMOS star sensor can be expressed as follows

(19) $$V_{c}=\left[\begin{array}{c} v_{x}\\ v_{y}\\ v_{z} \end{array}\right]=R_{rot}\cdot V_{0},$$

where $V_{0}=\left[0,0,1\right]$ denotes the initial vector of the star simulator in the rotary table coordinate system; $R_{rot}$ is the rotation matrix [27] that provides different angles for the simulated starlight and is represented as

(20) $$R_{rot}=\left[\begin{array}{ccc} 1 & 0 & 0\\ 0 & \cos\varphi_{1} & \sin\varphi_{1}\\ 0 & -\sin\varphi_{1} & \cos\varphi_{1} \end{array}\right]\left[\begin{array}{ccc} \cos\varphi_{2} & 0 & -\sin\varphi_{2}\\ 0 & 1 & 0\\ -\sin\varphi_{2} & 0 & \cos\varphi_{2} \end{array}\right],$$

where $\varphi_{1}$ and $\varphi_{2}$ denote the rotation angles of the external and middle frames of the rotary table, respectively. For simplicity, we assumed that the coordinate system of the 3CMOS star sensor aligned with the coordinate of the green channel. Under different directions of incident starlight, the centroid coordinate of the green-channel imaging star spot $\left(x_{g},y_{g}\right)$ can be expressed as

(21) $$\left\{\begin{array}{c} x_{g}=F_{g}\cdot \frac{v_{x}}{v_{z}}+\delta x_{g}+x_{0}^{g}\\ y_{g}=F_{g}\cdot \frac{v_{y}}{v_{z}}+\delta y_{g}+y_{0}^{g} \end{array}\right.,$$

the centroid coordinate of the red-channel imaging star spot $\left(x_{r},y_{r}\right)$ is given by

(22) $$\left[\begin{array}{c} x_{r}\\ y_{r} \end{array}\right]=\left[\begin{array}{cc} \cos\theta_{rg} & \sin\theta_{rg}\\ -\sin\theta_{rg} & \cos\theta_{rg} \end{array}\right]\left[\begin{array}{c} F_{r}\cdot \frac{v_{x}}{v_{z}}+\delta x_{r}\\ F_{r}\cdot \frac{v_{y}}{v_{z}}+\delta y_{r} \end{array}\right]+\left[\begin{array}{c} x_{0}^{r}\\ y_{0}^{r} \end{array}\right],$$

and that of the green-channel imaging star spot $\left(x_{b},y_{b}\right)$ is obtained by

(23) $$\left[\begin{array}{c} x_{b}\\ y_{b} \end{array}\right]=\left[\begin{array}{cc} \cos\theta_{bg} & \sin\theta_{bg}\\ -\sin\theta_{bg} & \cos\theta_{bg} \end{array}\right]\left[\begin{array}{c} F_{b}\cdot \frac{v_{x}}{v_{z}}+\delta x_{b}\\ F_{b}\cdot \frac{v_{y}}{v_{z}}+\delta y_{b} \end{array}\right]+\left[\begin{array}{c} x_{0}^{b}\\ y_{0}^{b} \end{array}\right].$$

The rotary table was rotated at various angles to ensure that the star spots covered the entire image plane of the detector within the FOV. At each position, the 3CMOS star sensor acquired and processed the star images to extract the centroid coordinate of each star spot. To reduce the influence of position noise, this process was repeated at least 10 times in this experiment, and the average value of the centroid coordinates was used as the calibration point. After the rotary table rotated n different positions, we obtained n groups of calibration point data.

### 3.2. Parameter Calculation

To solve the problem of two nonlinear functions involving ${f_{x}(u}_{bg\;})$ and ${f_{y}(u}_{rg})$, this study adopted a nonlinear least-squares iteration approach, which consisted of two main steps. Taking $u_{bg\;}$ as an example, the detailed parameter solving process is introduced as follows.

In step 1, we determined the initial values of the parameters that were close to the true values, thereby preventing the least-squares method from falling into a local extremum. To achieve this, the initial value of the scale factor $k_{bg}$ was set to 1 based on the actual imaging conditions of the camera. The initial value of $\theta_{bg}$ was estimated using the centroid distribution of the star spots imaged at specific rotation angles of the rotary table. Specifically, by keeping the external frame of the rotary table fixed at the zero position and rotating only the middle frame, the centroids of blue-channel and green-channel imaging star spots could be fitted with two straight lines, denoted as $l_{g}$ and $l_{b}$, respectively. The angle between two lines $l_{g}$ and $l_{b}$ was then considered as the initial value of $\theta_{bg}$. Furthermore, the centroid of the imaging star spot was considered as the value of the principal point for each channel when the rotary table was at the zero position. Then, the initial value of ($∆x_{bg},∆y_{bg}$) could be estimated by using Equation (14).

In step 2, with the estimated initial values and the constraint condition ${(\cos\theta_{bg})}^{2}$ + ${(\cos\theta_{bg})}^{2}$ = 1, each iteration of $u_{bg\;}\;$ was obtained by using the following expression

(24) $$u_{bg}^{\left(m+1\right)}=u_{bg}^{\left(m\right)}-(J^{T^{\left(m\right)}}J^{\left(m\right)}{)}^{-1}J^{T^{\left(m\right)}}T^{\left(m\right)},$$

where *m* represents the iteration time, **J** is a 2n × 4 matrix consisting of the sensitive matrixes **A** and **B**, and **T** is a 2n × 1 matrix consisting of the estimated deviations $∆x_{n}$ and $∆y_{n}$; *n* is the group number of the calibration points.

(25) $$J={\left[A_{1}\cdots A_{n}\;B_{1}\cdots B_{n}\right]}^{T},\;\;\;T={\left[∆x_{1}\cdots ∆x_{n}\;∆\cdots ∆y_{n}\right]}^{T},$$

where $\left\{\begin{array}{c} A=[\frac{\partial f_{x}}{\partial k_{bg}}\;\frac{\partial f_{x}}{\partial \theta_{bg}}\;\frac{\partial f_{x}}{\partial ∆x_{bg}}\;\frac{\partial f_{x}}{\partial ∆y_{bg}}]\\ B=[\frac{\partial f_{y}}{\partial k_{bg}}\;\frac{\partial f_{y}}{\partial \theta_{bg}}\frac{\partial f_{y}}{\partial ∆x_{bg}}\;\frac{\partial f_{y}}{\partial ∆y_{bg}}] \end{array}\right.$, $\left\{\begin{array}{c} ∆x_{n}=x_{g}^{\left(n\right)}-f_{x}^{\left(n\right)}(u_{bg})\\ ∆y_{n}=y_{g}^{\left(n\right)}-f_{y}^{\left(n\right)}(u_{bg}) \end{array}\right.$.

At the end of each iteration, the optimized values of the four parameters were obtained and updated as initial values for the next iteration. When the cost function was reduced to its minimum, the optimized solution of the parameter vector was considered to be obtained.

In the specific solving process, the following strategies were employed to ensure the precision of parameter optimization: (1) A two-step optimization method: we first optimized the scale factor $k_{bg}$ and the rotation angle $\theta_{bg}$. Subsequently, the values obtained in the first step were used as initial values when optimizing all four parameters $k_{bg,\;}\theta_{bg}$, $∆x_{bg},\;and\;∆y_{bg}$ together. (2) According to the configuration data provided by the camera manufacturer, we introduced random initial values within a certain range to complete the optimization process. Through the above series of steps, we could obtain the optimal solution for the parameter vector $u_{bg\;}\;\mathrm{and}\;u_{rg}$.

## 4. Experiments and Discussion

During the practical calibration process, it is necessary to first extract the centroids of imaging star spots of the 3CMOS star sensor. However, this extraction process can be affected by imaging noise, the algorithms used for extraction, and other random factors, resulting in centroid positioning errors. Therefore, a simulation experiment was performed to validate the effectiveness of the proposed calibration method under certain star position errors. Subsequently, a registration experiment on real star images was performed, with these images acquired using the AP-3200T prism camera [28] manufactured by JAI company.

### 4.1. Numerical Simulation

The parameters in the simulations were set according to the mentioned prism camera, where the FOV was 16° × 12°, the number of pixels in the image detector was 2064 pixels × 1544 pixels, and the element was 3.45 μm × 3.45 μm. The main imaging parameters for the three channels are listed in Table 1.

Using the imaging coordinate of the green channel as a reference, the true values of the transformation model parameters calculated according to Table 1 are shown in Table 2.

**Figure 4:**
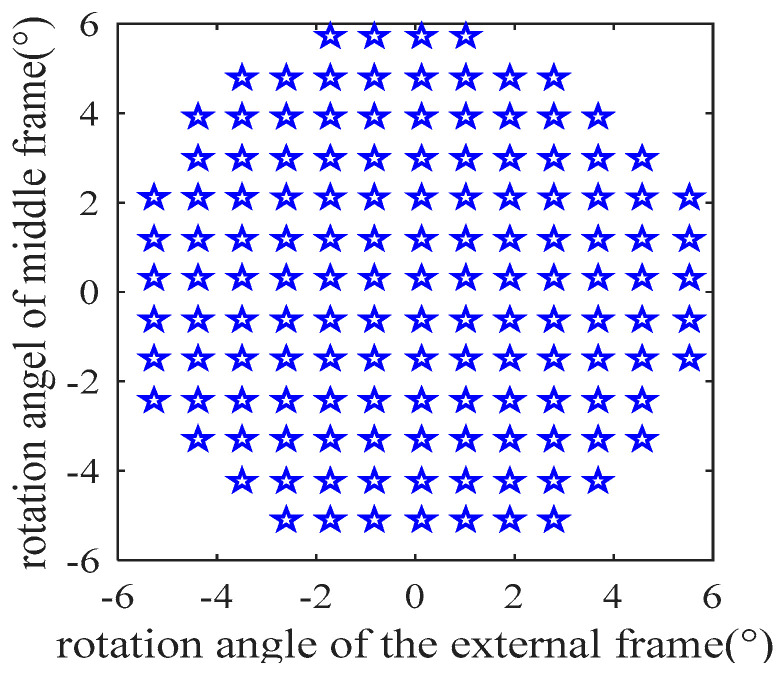
Rotation angle distribution of rotary table for generating star centroid coordinates of three channels.

In the simulation, we first generated a uniform 137 rotation angles, distributed over the range of (−8°, +8°) × (−6°, +6°) with a step size of 0.9°, as shown in Figure 4.

For each rotation angle, the centroid coordinates of green, red, and blue channels were generated using the imaging Equations (21)–(23). To simulate the star location errors and make the simulation close to the real calibration conditions, a Gaussian noise with a mean of 0 and a standard deviation of 0.05 was added to the generated star spot centroid coordinates. Subsequently, the steps described in Section 3.2 were carried out to compute the transformation model parameters. The results obtained are listed in Table 3.

### 4.2. Result Discussion

A comparison of Table 2 and Table 3 shows a slight disparity between the solved values and actual values when the noise was added to the star position. To evaluate the accuracy of the proposed calibration method, a total of 52 test point data were randomly generated, following the same method as the calibration data.

By substituting the test points into the transformation models (17) and (18) with the calibrated parameters shown in Table 3, we obtained the transformed centroid coordinates of the test points, denoted as $\left(x_{t},\;y_{t}\right)$. Taking the centroid coordinates of the green-channel test points $(x_{g}$,$y_{g})$ as the reference, this experiment evaluated the calibration accuracy by using the root mean square error (*RMSE*); *N* denotes the number of test points.

(26) $$RMSE=\sqrt{\frac{1}{N}(\sum_{i=1}^{N}{\left\|(x_{t},y_{t}{)}^{T}-(x_{g},y_{g}{)}^{T}\right\|}^{2}},$$

The statistical RMSE of the red channel relative to the green channel before the transformation was 1.0286 pixels. After the transformation, this value reduced to 0.0726 pixels. Similarly, the RMSE of the blue channel relative to the green channel before the transformation was 1.1557 pixels, which reduced to 0.0694 pixels after the transformation. The transformed RMSE errors, calculated using the transformation parameters obtained from Table 3, were approximately equal to the level of noise added to the positional noise. The above results prove that the proposed calibration method can be used to extract transformation model parameters based on the established transformation model and optimization strategy.

### 4.3. Registration Experiment of the 3CMOS Star Sensor

**Figure 5:**
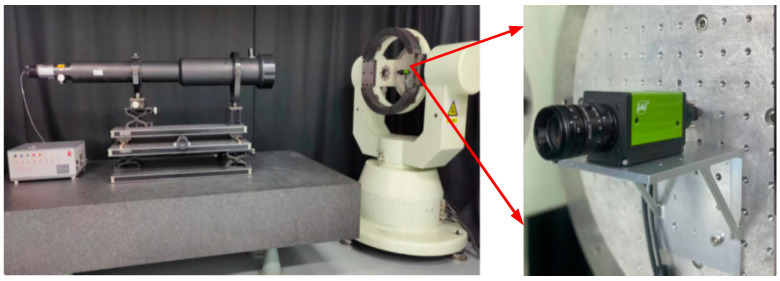
Calibration data acquisition setup.

The data acquisition setup, illustrated in Figure 5, comprised a single-star simulator and a high-precision rotary system, with the 3CMOS star sensor fixed on the middle frames of the turntable. A single-star simulator was used to emit simulated starlight of different spectral bands and magnitudes. In this registration experiment, the star simulator emitted starlight with a wavelength range of 400–700 nm at a magnitude of 4MV. The camera parameters were as follows: the FOV was 16° × 12°, and the estimated focal length was 25 mm. The pixel number of the image detector was 2064 × 1544 with an element of 3.45 μm × 3.45 μm, as described in [27]. The detector’s detection wavelengths for the blue, green, and red channels were 400–500 nm, 490–590 nm, and 580–680 nm, respectively. The rotary table provided a position accuracy of 0.5 arcsecs. When the turntable rotates the external and middle frames according to the set trajectory, it can provide starlight incidence at different angles, ensuring that the imaging star points cover the entire image plane within FOV. The exposure time for the star sensor was set to 100 ms. Under these configurations, an number of calibration data were collected by this device. To avoid interference from stray light, the data acquisition was performed in a dark-room environment.

Similar to the simulation process, the calculation parameters of the 3CMOS star sensor were determined by using the initial parameter values set to $u_{bg\;}$= (1, 0°, 0,0), $u_{rg}$= (1, 0°, 0,0), which were obtained by performing the initial value estimation method described in Section 3.2. In addition, to verify the effectiveness of distortion simplification analysis in this model, the imaging parameter focal length and distortion coefficient for each channel were estimated using a similar nonlinear least-squares method based on Equation (2). These results are also listed in Table 4.

After obtaining the calibrated parameters, a total of 52 group test data were collected at random positions to evaluate the registration accuracy. The statistical results show that, before the transformation, the RMSE of the red channel with respect to the green channel was 1.4027 pixels and 1.3986 pixels in the x and y directions, respectively. After the transformation, these values significantly decreased to 0.2135 pixels and 0.2089 pixels in the x and y directions, and the equivalent pointing errors were 6.0692 arcsecs and 5.9385 arcsecs. Similarly, for the blue channel relative to the green channel, the RMSE before the transformation was 1.3584 pixels and 1.3416 pixels in the x and y directions. However, after the transformation, these values reduced to 0.2146 pixels and 0.2097 pixels in the x and y directions, corresponding to the equivalent pointing errors of 6.1035 arcsecs and 5.9614 arcsecs. The level of equivalent pointing errors was approximately equal to the starlight pointing error measured by the monochromatic star sensor in the laboratory. These results suggest that the registration accuracy is satisfactory, effectively ensuring the precision of subsequent operations for the 3CMOS star sensor. In the practical registration process, the registration accuracy may also be influenced by the work environment and random star position noise. The calibrated focal length and distortion coefficient results in Table 4 indicate that the simplified process of the distortion relationship between two adjacent channels in this model is reasonable.

## 5. Conclusions

In this study, we introduced a star image registration method for 3CMOS star sensors, which lays a foundation for subsequent high-precision star image fusion and star centroid extraction.

The main contributions of this work include the establishment of registration models and the development of techniques for estimating model parameters. Our proposed method takes the imaging star position as the feature point to perform image registration. By carefully analyzing the effect of inconsistent three-channel imaging parameters on the position of feature points, we developed a registration mode that can transform the star images from different channels into the reference coordinate system. This model simplifies the distortion relationship between two adjacent channels based on the imaging properties of 3CMOS star sensors, making the registration process straightforward and feasible. Additionally, our parameter estimation scheme based on linear least squares facilitates high-precision parameter calibration, ensuring the accurate registration of star images.

The experimental results demonstrate the effectiveness of our proposed registration method; it achieves subpixel alignment accuracy, even under real imaging conditions with some degree of position noise. This level of accuracy can guarantee the accuracy of subsequent operations. Our method provides an image alignment solution for similar imaging systems, such as multi-channel imaging cameras. Future work will focus on researching star image fusion technology to extract more accurate star centroids based on the spectral radiation characteristics of stars, enabling the pointing accuracy of starlight measured by 3CMOS star sensors to compete with that of monochromatic star sensors.

## Figures and Tables

**Table 1:** Main imaging parameters of 3CMOS star sensor in simulation.

Parameter	Red Channel	Green Channel	Blue Channel
Focal length (mm)	25.0601	25.0000	24.9500
Principal point (pixel)	(1033.9, 774.3)	(1032, 772)	(1030.6, 770.4)
Radial distortion	5.7 × 10^−5^	4.9 × 10^−5^	3.6 × 10^−5^

**Table 2:** The true value of transformation parameters in the simulation.

Parameter	Red Channel	Blue Channel
Focal length scale factor	0.9976	1.0020
Rotation angle (°)	0.07	−0.05
Translation (pixel)	(0.3, 0.5)	(−0.4, −0.6)

**Table 3:** Simulation results when noise was added to the star position.

Parameter	Red Channel	Blue Channel
Focal length scale factor	0.9973	1.0018
Rotation angle (°)	0.0698	−0.0501
Translation (pixel)	(0.3012, 0.5024)	(−0.4032, −0.5986)

**Table 4:** Parameter calibration results for the 3CMOS star sensor.

Parameter	Red Channel	Green Channel	Blue Channel
Focal length scale factor	0.9996	-	1.0012
Rotation angle (°)	0.0219	-	−0.0351
Translation (pixel)	(−0.9178, 0.9364)	-	(−0.9256, 1.0961)
Focal length (mm)	25.0304	25.0237	25.0134
Radial distortion	3.4166 × 10^−5^	3.4152 × 10^−5^	3.4147 × 10^−5^

## Data Availability

Data are contained within the article.
